# An in vitro splicing assay reveals the pathogenicity of a novel intronic variant in *ATP6V0A4* for autosomal recessive distal renal tubular acidosis

**DOI:** 10.1186/s12882-017-0774-4

**Published:** 2017-12-04

**Authors:** Tomohiko Yamamura, Kandai Nozu, Yuya Miyoshi, Keita Nakanishi, Junya Fujimura, Tomoko Horinouchi, Shogo Minamikawa, Nobuo Mori, Rika Fujimaru, Koichi Nakanishi, Takeshi Ninchoji, Hiroshi Kaito, Taniguchi-Ikeda Mariko, Ichiro Morioka, Masafumi Matsuo, Kazumoto Iijima

**Affiliations:** 1 Department of Pediatrics, Kobe University Graduate School of Medicine, 7-5-1 Kusunokicho, Chuo, Kobe, Hyogo 6500017 Japan; 2 Nakano Children’s Hospital, 4-13-17 Shinmori, Asahi, Osaka, 5350022 Japan; 3 Department of Pediatrics, Osaka City General Hospital, 2-13-22 Miyakojimahondori, Miyakojima, Osaka, 5340021 Japan; 4 Department of Child Health and Welfare (Pediatrics), Graduate School of Medicine, University of the Ryukyus, 207 Uehara, Nishihara, Nakagami 9030125 Japan; 5 Department of Physical Therapy, Faculty of Rehabilitation, Kobe Gakuin University, 518 Arise, Ikawadani, Nishi, Kobe, 6512180 Japan

**Keywords:** Autosomal recessive distal renal tubular acidosis, *ATP6V0A4*, Minigene, Splicing assay

## Abstract

**Background:**

Autosomal recessive distal renal tubular acidosis (dRTA) is a rare hereditary disease caused by pathogenic variants in the *ATP6V0A4* gene or *ATP6V1B1* gene, and characterized by hyperchloremic metabolic acidosis with normal anion gap, hypokalemia, hypercalciuria, hypocitraturia and nephrocalcinosis. Although several intronic nucleotide variants in these genes have been detected, all of them fell in the apparent splice consensus sequence. In general, transcriptional analysis is necessary to determine the effect on function of the novel intronic variants located out of splicing consensus sequences. In recent years, functional splicing analysis using minigene construction was used to assess the pathogenicity of novel intoronic variant in various field.

**Methods:**

We investigated a sporadic case of dRTA with a compound heterozygous mutation in the *ATP6V0A4* gene, revealed by next generation sequencing. One variant was already reported as pathogenic; however, the other was a novel variant in intron 11 (c.1029 + 5G > A) falling outside of the apparent splicing consensus sequence. Expression of *ATP6V0A4* was not detected in peripheral leukocytes by RT-PCR analysis. Therefore, an in vitro functional splicing study using minigene construction was conducted to analyze the splicing pattern of the novel variant.

**Results:**

A minigene assay revealed that the novel intronic variant leads to a 104 bp insertion immediately following exon 11. In addition, this result was confirmed using RNA extracted from the patient’s cultured leukocytes.

**Conclusion:**

These results proved the pathogenicity of a novel intronic variant in our patient. We concluded that the minigene assay is a useful, non-invasive method for functional splicing analysis of inherited kidney disease, even if standard transcriptional analysis could not detect abnormal mRNA.

**Electronic supplementary material:**

The online version of this article (10.1186/s12882-017-0774-4) contains supplementary material, which is available to authorized users.

## Background

Renal tubular acidosis (RTA) is characterized by normal serum anion gap or hyperchloremic metabolic acidosis and four types of RTA can be distinguished on the basis of clinical, pathophysiological, and molecular differences. [1, 2]. Hereditary distal renal tubular acidosis (dRTA) is caused by mutations in genes that encode three proteins expressed in α-intercalated cells of the collecting duct, namely, the B1 or A4 subunits of V-ATPase and the kidney Cl^−^ /HCO^−^ exchanger (kAE1) [2–5]. *ATP6V1B1* and *ATP6V0A4* encode the B1 and a4 subunits of the V-ATPase, respectively. Pathogenic variants in these genes interrupt the function of V-ATPase and cause autosomal recessive dRTA and sensorineural hearing loss.

In recent years, it was reported that next generation sequencing (NGS) is a useful diagnostic tool of the genetic analysis for dRTA and many pathogenic variants including intronic variants have been detected so far [6]. However, fewer than 10 intronic variants have been identified in the *ATP6V0A4* gene and all these variants are located in apparent splicing consensus sequences (HGMD Professional 2016.4) [7]. In general, transcript analysis is necessary for variants located outside of the splicing consensus sequence. However, this technique is difficult for dRTA because the *ATP6V0A4* transcript expression level is very low in peripheral blood leukocytes, usually the only samples available from patients.

Recently, functional splicing analysis using minigene construction has been used to investigate novel intronic variants in various inherited diseases and revealed its usefulness [8–12]. However, in the field of inherited kidney diseases, there is very little information about this minigene assay [13, 14].

## Methods

A one-year-old Japanese boy was referred to our hospital for further evaluation of failure to thrive and suspected renal tubular acidosis (RTA) by the family doctor. Blood examination showed hyperchloremic non-gap metabolic acidosis and hypokalemia (chloride: 110 meq/L; blood pH: 7.25; bicarbonate: 11.5 mmol/L; potassium: 3.1 meq/L). Urinalysis showed high urine pH (7.0), positive urine anion gap (15 mmol/L), hypercalciuria, hyperkaliuria and low molecular proteinuria (calcium/creatinine ratio: 0.38 g/gcre (normal: below 0.2 g/gcre); potassium/creatinine ratio 10.5 (normal: below 2); beta-2-microgloblin: 79,639 μg/L (normal: below 400μg/L)). The calculated transtubular potassium gradient (TTKG) was 5.5 and the fractional excretion of potassium (FEK) was 9.8%. Abdominal ultrasonography detected bilateral renal calcinosis. Furosemide and NaHCO_3_ load test indicated that clinical diagnosis was dRTA. Audiometry screening test did not detect sensorineural deafness.

After obtaining informed consent from the parents of the proband, we performed targeted sequencing using next generation sequencing (NGS). NGS samples were prepared using a HaloPlex Target Enrichment System Kit (Agilent Technologies, Santa Clara, CA, USA) according to the manufacturer’s instructions to capture 17 genes (Additional file 1: Table S1), including genes associated with renal tubular disorder. Amplified target libraries were sequenced using MiSeq (Illumina, San Diego, CA, USA) and analyzed with SureCall (v.3.0; Agilent Technologies). The ATP6V0A4 reads were mapped to the human reference sequence NM_020632.2.

In this study, hybrid minigene constructs were created by inserting a test sequence fragment consisting of exon 11 and exon 12 and its flanking introns into the multicloning site within an intervening intron between two exons (exon A and B) of the minigene construct (H492), built in the pcDNA 3.0 mammalian expression vector (Invitrogen, Carlsbad, CA) (Additional file 1: Figure S1) [14]. These hybrid minigenes were transfected into HEK293T cells and HeLa cells for splicing assays as described elsewhere [10, 11]. Both HEK293T and HeLa cells were obtained from the Cell Bank, Riken Bio Resource Center (Tsukuba, Japan). Total RNA was reverse-transcribed into cDNA and the PCR was performed using specific primers as previously described [10] (Additional file 1: Figure S1). PCR products were analyzed by means of electrophoresis and direct sequencing.

In addition, patient’s leukocyte culture was performed to increase the total RNA amount for detecting splicing abnormalities. The detailed method of cultured leukocyte is as follows; 8.5 mL of patient’s peripheral blood is mixed to 1.5 mL of Acid Citrate Dextrose solution (Becton-Dickinson & Company, Franklin Lakes, NJ) at room temperature. Transfer a blood and Acid Citrate Dextrose solution mix into a 50 ml tube and dilute blood with an equal volume of PBS and mix gently. Add 5 mL of Ficoll-Paque PREMIUM (GE Healthcare, UK) to a 15 mL tube. Gently overlay 5 mL of the diluted blood onto 5 mL of Ficoll without disturbing the interface. Centrifuge at 400×g for 30 min at 25 °C and carefully collect the whitish layer in the upper-middle interface into a 15 mL tube. Add 6 times of volume of PBS and mix gently and centrifuge at 200×g for 10 min at 25 °C. Aspirate the supernatant and add 2 mL of KBM-502 medium (KOHJIN BIO, JP) to the pellet. Incubate cells at 5% CO_2_ incubator at 37 °C for 1 week and correct total RNA.

## Results

### Mutational analysis

**Fig. 1:**
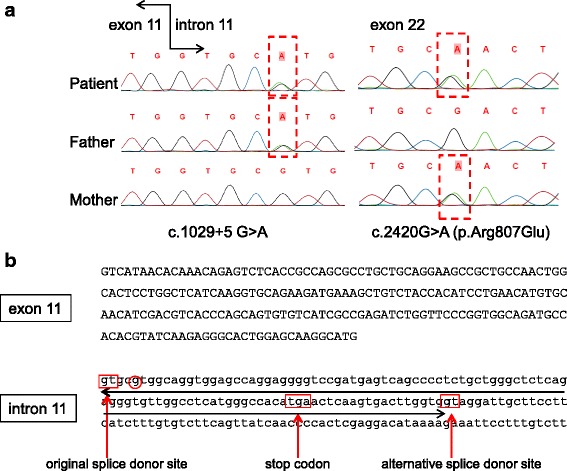
Mutations detected in the *ATP6V0A4* gene. **a** Left; A heterozygous single-base substitution of G to A in intron 11 (C.1029 + 5A > G) was detected in the paternal allele. Right; A heterozygous single-base substitution of G to A in exon 22 (c.2420 G > A) was detected in the maternal allele. **b** Normal sequence of exon 11 and intron 11 in the *ATP6V0A4* gene. The original splice donor site was interrupted by the mutation (c.1029 + 5G > A) and the sequence of “GT” 104 bp later was used preferentially as an alternative splice donor site. The insertion following exon 11 is underlined and results in a stop codon within the insertion

Targeted sequencing and direct sequencing revealed a novel heterozygous variant of one base substitution at the 5th base of intron 11(c.1029 + 5G > A) and a previously reported variant of one base substitution in exon 22 (c.2420G > A, p.Arg807Gln [15]) in the *ATP6V0A4* gene. The variants were derived from the paternal and maternal alleles, respectively (Fig. 1a). RNA was extracted from peripheral blood leukocytes for RT-PCR analysis, however the *ATP6V0A4* transcript failed to amplify because of its low expression.

### In vitro splicing assay

**Fig. 2:**
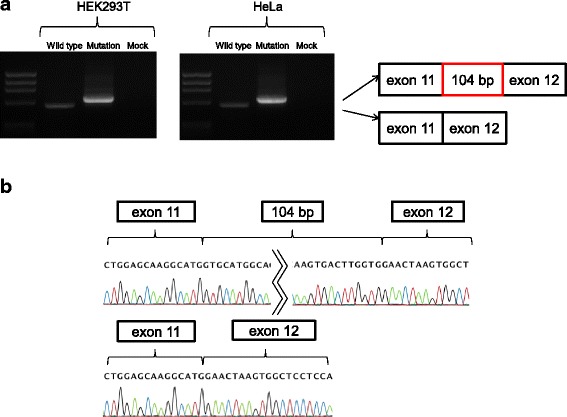
In vitro splicing assay using hybrid minigene construct. **a** RT-PCR amplified products of hybrid minigene transcripts. As shown in the gel file, a comparatively small band was present in the wild type construct both in HEK293T and HeLa cells. In contrast, in the minigene derived from the patient sequence, only a relatively large band was detected in both cell lines, which corresponds to a 104 bp insertion between exon 11 and exon12. **b** The RT-PCR product containing the 104 bp insertion between exon 11 and exon 12 was obtained from the minigene encoding the intronic mutation (b top). On the other hand, the product containing exon 11 and exon 12 could be obtained from the hybrid minigene encoding the wild type genomic DNA (b bottom)

The RT-PCR product containing the expected exon 11 - exon 12 sequence between the cassette exons A and B was obtained from the minigene encoding the wild-type sequence (Fig. 2a-b). In contrast, the product from the hybrid minigene containing patient sequence with the intronic variant showed a 104 bp sequence insertion between exon 11 and exon 12.

### RT-PCR using RNA extracted from cultured leukocytes

**Fig. 3:**
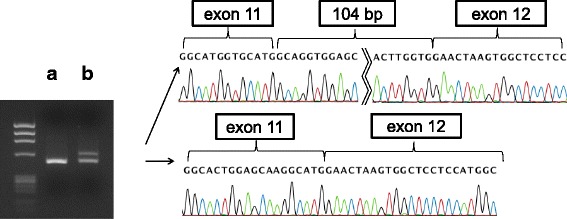
Direct sequencing results of cDNA after PCR. The control leukocyte sample shows only one normal band (**a**), the sample extracted from lymphocyte culture of the patient (**b**) shows two bands, one the same size as the control sample and the other including a 104 bp insertion sequence

After finding the splicing abnormality using the minigene construct, we used RT-PCR analysis on RNA extracted from patient cultured lymphocytes to confirm the result. RT-PCR showed two bands: one the same size as the control, and the other larger, consistent with the 104 bp product detected in the minigene assay (Fig. 3). Sequencing of the larger product confirmed the 104 bp insertion following exon 11. This insert sequence created a stop codon.

## Discussion

To assess the biological effect of intronic variants that do not fall in splicing consensus sequences, transcript analysis is necessary. However, there are several reasons why this may not be successful. First, abnormal transcripts can be hard to amplify by RT-PCR because of the influence of nonsense-mediated mRNA decay (NMD) for the products of truncating variants. Second, transcripts extracted from tissues other than blood leukocytes are not always available in various diseases and targeted transcript expression may be low in leukocytes. To resolve these problems, we conducted an in vitro splicing assay using hybrid minigene construction, which revealed an alternative splice donor site that was used in preference to the original site (Fig. 1b). This finding indicated that the c.1029 + 5A > G variant was pathogenic. To confirm this result, we used mRNA extracted from cultured leukocytes and, fortunately, we could detect the same 104 bp insertion.

We were able to detect the abnormal transcript in the patient’s cultured leukocytes in this study. However, this method takes a lot of effort, including obtaining fresh samples, which is not possible for all cases. Although renal biopsy specimens could be used as a source of mRNA in inherited kidney diseases, renal biopsy is invasive and not necessary for most inherited kidney diseases, including dRTA. Alternatively, urine sediments can be used for genetic analysis in inherited kidney diseases [14, 16]. However, in this case, we also failed to obtain mRNA from patient’s urine because of polyuria, a symptom of RTA, which results in diluted urine.

Here we report, for the first time, the use of an in vitro splicing assay using minigene construction, in the characterization of a mutation in dRTA, although this assay has previously been conducted for several diseases, including some inherited kidney diseases [10–14, 17, 18]. Some reports used this analysis because they could not obtain mRNA from kidney specimens [17]. This time, we could confirm the splicing abnormality using both the patient’s sample and minigene construction.

In silico analysis is generally used to estimate the pathogenicity of novel intronic variants. In this case, we tried to predict the alternative splice donor site using Human Splicing Finder 3.0 (http://www.umd.be/HSF3/) [19]; however, this variant was predicted as having “probably no impact” on splicing (Additional file 1: Figure S2). This shows that in silico analysis does not always predict accurately. Therefore, the in vitro functional splicing assay using a minigene is an attractive tool for cases such as the one described here.

## Conclusions

In conclusion, our study demonstrates that the minigene assay is a useful, non-invasive method for functional splicing analysis of inherited kidney disease, even if standard transcriptional analysis could not detect abnormal mRNA. This analytical assay could be adapted for other inherited kidney diseases.

## Additional file

Additional file 1: Table S1. List of genes analyzed by target sequencing in this study. **Figure S1.** Diagram of hybrid minigene construct. **Figure S2.** The result of alternative splicing pattern prediction using Human Splicing Finder Ver.3.0. (DOCX 296 kb)

## Abbreviations

dRTA: Distal renal tubular acidosis
RT-PCR: Reverse-transcription PCR
