# Supplementary material for: An in vitro splicing assay reveals the pathogenicity of a novel intronic variant in ATP6V0A4 for autosomal recessive distal renal tubular acidosis
Source: BMC Nephrol. 2017 Dec 4;18:353. doi: 10.1186/s12882-017-0774-4 (PMC5716019; doi:10.1186/s12882-017-0774-4)
Supplement: Additional file 1: Table S1. — List of genes analyzed by target sequencing in this study. Figure S1. Diagram of hybrid minigene construct. Figure S2. The result of alternative splicing pattern prediction using Human Splicing Finder Ver.3.0. (DOCX 296 kb) [file 12882_2017_774_MOESM1_ESM.docx]

**Supplementary Table 1. List of genes analyzed by target sequencing in this study**

| **Gene** | | | **Gene** | | | **Gene** | | |
| --- | --- | --- | --- | --- | --- | --- | --- | --- |
| 1 | *ATP6V0A4* | All exons | 7 | *HNF4A* | All exons | 13 | *SLC34A1* | All exons |
| 2 | *ATP6V1B1* | All exons | 8 | *KLHL3* | All exons | 14 | *SLC4A1* | All exons |
| 3 | *CA2* | All exons | 9 | *NR3C2* | All exons | 15 | *SLC4A4* | All exons |
| 4 | *CTNS* | All exons | 10 | *SCNN1A* | All exons | 16 | *WNK1* | All exons |
| 5 | *CUL3* | All exons | 11 | *SCNN1B* | All exons | 17 | *WNK4* | All exons |
| 6 | *EHHADH* | All exons | 12 | *SCNN1G* | All exons |  |  |  |

**Supplementary Figure 1. Diagram of hybrid minigene construct**

A minigene (H492) was constructed to encode two cassette exons (A and B) and an intervening sequence containing a multicloning site. The minigene contained a cytomegalovirus (CMV) enhancer–promoter and a bovine growth hormone gene (BGH) polyadenylation signal (black boxes) for complete synthesis of mRNA. The primers used in the RT-PCR assay are represented by arrows.

**Supplementary Figure 2. The result of alternative splicing pattern prediction using Human Splicing Finder Ver.3.0**

*In silico* analysis using Human Splicing Finder 3.0 (http://www.umd.be/HSF3/) predicted that a novel mutation in intron 11 (c.1029+5G>A) of *ATP6V0A4* gene has no probable impact on splicing.
